# Significance of LL-37 on Immunomodulation and Disease Outcome

**DOI:** 10.1155/2020/8349712

**Published:** 2020-05-16

**Authors:** Binbin Yang, David Good, Tamim Mosaiab, Wei Liu, Guoying Ni, Jasmine Kaur, Xiaosong Liu, Calvin Jessop, Lu Yang, Rushdi Fadhil, Zhengjun Yi, Ming Q. Wei

**Affiliations:** ^1^School of Medical Laboratory, Key Laboratory of Clinical Laboratory Diagnostics in Universities of Shandong, Weifang Medical University, Weifang 261053, China; ^2^School of Medical Science & Menzies Health Institute Queensland, Griffith University, Gold Coast, Qld 4215, Australia; ^3^School of Allied Health, Australian Catholic University, Brisbane, Qld 4014, Australia; ^4^Institute for Glycomics, Griffith University, Gold Coast, Qld 4215, Australia; ^5^The First Affiliated Hospital/School of Clinical Medicine, Guangdong Pharmaceutical University, Guangzhou 510006, China; ^6^School of Health and Sport Sciences, University of the Sunshine Coast, Maroochydore DC, Qld 4558, Australia; ^7^Cancer Research Institute, First People's Hospital of Foshan, Foshan 528000, China

## Abstract

LL-37, also called cathelicidin, is an important part of the human immune system, which can resist various pathogens. A plethora of experiments have demonstrated that it has the multifunctional effects of immune regulation, in addition to antimicrobial activity. Recently, there have been increasing interest in its immune function. It was found that LL-37 can have two distinct functions in different tissues and different microenvironments. Thus, it is necessary to investigate LL-37 immune functions from the two sides of the same coin. On the one side, LL-37 promotes inflammation and immune response and exerts its anti-infective and antitumor effects; on the other side, it has the ability to inhibit inflammation and promote carcinogenesis. This review presents a brief summary of its expression, structure, and immunomodulatory effects as well as brief discussions on the role of this small peptide as a key factor in the development and treatment of various inflammation-related diseases and cancers.

## 1. Introduction

Antimicrobial peptides (AMPs) are important elements of the immune system which are capable of combating a broad spectrum of organisms and transformed or cancerous cells [1, 2]. Mammalian AMPs belong to the defensin and cathelicidin families. So far, there is a unique cathelicidin peptide found in 1995 and called human cationic antimicrobial peptide (hCAP18) [2]. Its active part starts with double leucine and consists of 37-amino acids at the C-terminus, so which is called LL-37. Not only human but also only one cathelicidin the analogue of human cathelicidin was found in mice named cathelicidin-related antimicrobial protein (CRAMP).

This small number of AMPs is expressed in some cell types that may encounter pathogens. Cathelicidin is involved in the innate immune system; after infection, LL-37 bridges the innate and acquired immunity through recruiting immune cells to the infected site and stimulates and/or modulates adaptive immunity through specific activation of the receptors of the immune cells as well [3]. Moreover, LL-37 regulates the production of chemokines and pro- and anti-inflammatory cytokines, in order to maintain the fine balances between pro- and anti-inflammatory responses. This ability to maintain equilibrium plays a very important role in resisting pathogens while maintaining the stability of the immune system. If defects in the expression or processing of LL-37 break this balance, it will result in abnormalities of the body.

The purpose of this review is to offer a concise general view of the expression, structure, and immunomodulatory effects of antimicrobial peptides LL-37 on immunocompetent cells and briefly discuss the role of this small peptide as a key factor in the development and treatment of various inflammation-related diseases and cancers.

## 2. Structure

The human cathelicidin preprotein hCAP18, which is encoded on chromosome 3p21.3 [2], the genes that consist of 4 exons and 3 introns, is cleaved into active type LL-37 by protease-3 extracellularly under specific conditions [4]. The sequence of LL-37 is LLGDFFRKSKEKIGKEFKRIVQRIKDFLRNLVPRTES. At neutral pH, this peptide is amphipathic and cationic which net charge is +6 [5]. Although a state of random coil in pure water, in solutions with millimolar concentrations of salts or membrane-like environment, little cathelicidin peptide forms a *α*-helical structure with cationic amphipathic [1]. Conditions encouraging tend to favor *α*-helical structure rely on the solution condition including NaCl concentration and pH, membrane-like environment, or higher peptide concentrations [5].

There are three parts of the linear cationic *α*-helix of LL-37 structure, two *α*-helices of N-terminus and C-terminus which are from residues 2~30 of peptide and one unstructured C-terminal tail that consists of the C-terminal residues 31 to 37 [6]. The cationic hydrophobic surface of LL-37 enables to interact with negatively charged elements, like bacterial cell walls, lipopolysaccharide (LPS), and nucleotide, because of being formed by four aromatic phenylalanine side chains and the border by predominately positively charged residues [7]. The N-terminal helix is responsible for peptide oligomerization, proteolytic resistance, chemotaxis, and hemolytic activity, while the C-terminal helix is involved in antipathogen effect [8]. Above these two structures are needed in peptide aggregation [9]. The C-terminal tail is the critical structure to form a tetramer of peptide, which facilitates its ability to activate effect cells and favors to interact with components of serum and the bacterial outer wall that result peptide sequestration and decrease its antipathogen potential [9].

## 3. Expression

LL-37 is expressed in many types of tissue cells such as keratinocytes, differentiated epithelial cells in the colon, airway, ocular surface, genitals, in eccrine glands, Brunner glands in the duodenum [10], myelocytes [11], mesenchymal stromal cells (MSCs) [12, 13], and cells of testes [11]. Expression in most epithelia is constitutive, while injury can induce peptide expression in keratinocytes, where the precursor localizes in granules of the superficial epidermis and partially resides in lamellar bodies [14]. Chakraborty et al. indicated that the constitutive expression in epithelial cells is regulated through cAMP-signaling pathways, and some complexes are required which are formed by cAMP-responsive element-binding protein (CREB) and activator protein 1 (AP-1) to bind to the cathelicidin peptide promoter sequence and induce transcription [15].

As an important part of the immune system, cathelicidin protein constitutive expression is produced in natural killer (NK) cells, neutrophils, T cells, and mast cells [10, 16]. As the first line cells confront the pathogens, neutrophils are the main source of this peptide. The neutrophil synthesizes inactive hCAP18 precursor and store in secondary particles. Active toll-like receptors (TLRs) by damage-associated molecular pattern molecules (DAMPs) or pathogen-associated molecular pattern molecules (PAMPs), and/or changes in cytokine types and levels, can promote cell to degranulate. Upon stimulation, the cathelicidin protein precursor will be degranulated and released extracellularly, where the active cathelicidin proteolytically processed by specific proteases 3 was unleashed [4]. Furthermore, monocytes, dendritic cells (DCs), and macrophages of the immune system are proved to express LL-37 [10]. Peripheral blood-derived cells are proved to express a gradient dose: high levels in neutrophils; low levels in lymphocytes, while different types of lymphocytes of low level produce the same amount, and the expression of monocytes is moderate [17].

Different factors by different incentives from the body and outside can influence this peptide expression, for example, interferon *γ* (IFN-*γ*), interleukin 6 (IL-6), glucocorticoids, transmigration across activated endothelium, bacterial exotoxins, certain bacteria, entitystat, and calcipotriol are found to be the downregulated factors [10], while the upregulation of expression was found to be tumor necrosis factor *α* (TNF-*α*), IL-17A, toll-like receptor agonists, insulin-like growth factor 1 (IGF-1), vitamin D receptor agonists, hormonal 1,25dihydroxyvitamin D, phenyl butyrate, sodium butyrate, MUC2 mucin, simvastatin, injury and wounding, and endoplasmic reticulum stress [10, 18, 19].

As this small peptide plays an important role involved in the innate immune system and adaptive immune system, overexpression or low expression will break the immune balance to cause some diseases. In the inflamed mucosa of ulcerative colitis (UC) and Crohn's disease (CD), the expression of LL-37 mRNA was reported that has increased significantly [20, 21]. These researches display that the TLR3 ligand, double-stranded RNA (mimicked by polyinosinic-polycytidylic acid (poly(I:C))), and induced LL-37 mRNA overexpress in colonic subepithelial myofibroblasts (SEMFs) that proved self-nucleic acids break innate tolerance [20, 21], while overexpressed LL-37 found in psoriatic epidermis activates DCs to produce cytokines like TNF-*α*, IL-23, and IL-17 and become an autoantigen which can trigger the T cell and adaptive immune system [22–24]. In autoimmune diseases, overexpression of LL-37 excessively exerts its immune regulation, thus destroying the homeostasis of the immune system. On the other hand, lower expression also causes more severe infections, for instance, periodontitis caused by bacteria [25], tuberculosis [26, 27], oral squamous cell carcinoma (OSCC), and so on. Interestingly, because of functions of LL-37 in different tissue are pleiotropic, some changes in the expression of LL-37 appear in different tumors. Overexpression is found in ovarian cancer, lung cancer, breast cancer, malignant melanoma, and prostate cancer, but gastrointestinal cancer (colon cancer and gastric cancer) and hematological malignancies were reported lower than normal [28].

## 4. Chemotaxis

As previously stated, the infected epithelial cells expressed LL-37 which can directly recruit immunocompetent cells, including neutrophils, monocytes, and DCs. While the neutrophils are attracted, that is the first line dealing with microbes, as a main source of cathelicidin neutrophils that continue to be released at the infected sites. Furthermore, LL-37 induce monocyte [29], fibroblasts [30], epithelial cells [29], and human airway smooth muscle (HASM) cells [31] to secrete IL-8, which further indirectly attracts immune cells, then these cells together with LL-37 against the infection. In addition, other chemokines which have the ability to attract more immunocompetent cells like neutrophils, monocytes, DCs, and T cells, that are released by the cells encounter infections upon treatment with LL-37, for instance, CCL4, CCL20, and C-X-C motif ligand (CXCL) 1 proved to be produced by primary monocytes [32], C-C motif ligand (CCL) 2 released by endothelial cells [33], CCL3 and CCL2 by mast cells [34], and a synergistic increase in CCL20, CXCL1, CXCL8 (IL-8), and CCL2 secretion upon LL-37 exposure to keratinocyte-fibroblast cocultures [35]. After the activation of the innate immunity system, antigen-presenting cells carry the antigen to the specific T cells. Then, the cells of the adaptive immune system are directly attracted [1]. Current researches show that LL-37 plays a chemotactic role from the initial stage of infection to the adaptive immune response, promotes the anti-infective inflammatory response, and plays a bridging role from innate immunity to adaptive immunity. Not only chemotactic to immune cells, previous studies have shown that LL-37 recruit multipotent mesenchymal stromal cell (MSC) migration to tumor through formyl peptide receptor (FPR) 2 [36]. Using anti-LL-37 antibody to neutralize LL-37 in vivo can notably partly decrease the implantation of MSCs into ovarian cancer modeled by OVCAR-3 ovarian cancer cells, causing suppression of tumor growth and breakdown of the fibrovascular network. These findings consistent with that LL-37 treatment enhanced the proliferation and migration of human adipose-derived stromal/stem cells (ASCs) [37]. It is indicated that the LL-37-mediated recruiting MSCs could promote tumor progression.

## 5. Immunomodulation

### 5.1. Neutrophils

Neutrophil constitutes an important part of the innate immune system and is the front line to resist bacterial infection. Pathogen identification and subsequent recruitment of granulocytes into the infected site are key factors for host defense against bacterial diseases. This process includes the recognition of PAMPs by host pattern recognition receptors (PRRS), and the production of a variety of proinflammatory cytokines and chemokines at the site of infection. These chemical attractants promote the recruitment of neutrophils to infection and inflammation sites and produce variable potent mediators, including chemokines, cytokines, colony-stimulating factors, fibrous factors, and angiogenic factors, and then ingest and kill invading microorganisms. The effective antibacterial activity of neutrophils is the synergistic action between high protein hydrolase and degrading enzyme, cation molecule and active oxygen, which enables the immune system to successfully protect the host from various bacterial pathogens [38]. Under steady-state conditions, neutrophils undergo structural (spontaneous) apoptosis to end their short life (about 4-5 days). After neutrophil necrosis or apoptosis, neutrophil extractor traps (NETs) will be formed in the inflammatory site, which can provide high concentration of antibacterial molecules in the local area and quickly control the infection of bacteria in vivo. However, in abnormal circumstances, such as the induction of abnormal somatic cells, pathogens, or cancer cells, the secondary necrosis of apoptotic neutrophils happened, and the release of active molecules is continuously produced, forming an abnormal number of NETs, damaging healthy host cells, causing inflammation expansion and tissue damage [39–41].

As the main source of LL-37, neutrophils, stimulated by TLR ligands, not only can release antimicrobial peptides but also influenced by antimicrobial peptides and change physiological functions. It was reported that LL-37 induces neutrophil migration and chemotaxis mediated via FPR molecules in vitro [42, 43]. But neutrophil chemotaxis induced by serum amyloid A (SAA) almost competes inhibited by LL-37 [44]. SAA is an acute phase response protein produced by the body, which can be used to determine the severity of infection and inflammation. When SAA rises, it indicates that the body has produced a wide range of inflammation. We think that LL-37 may play an important role in immune regulation, limit the further expansion of SAA-induced inflammation, and maintain the stability of the immune system. The same condition happened in sepsis-induced acute lung injury, LL-37 and its analogy sLL-37 through the focal adhesion kinase (FAK), extracellular signal-regulated kinase (ERK), and P38 pathways which inhibit neutrophil infiltration and migration after the severe infection [45]. Interestingly, the cathelicidin can depress the expression of C-X-C chemokine receptor type 2 (CXCR2), which is a neutrophil surface receptor that mediates neutrophil migration to the sites of inflammation [46, 47].

These two sides function as well as reflect regulation of LL-37 for neutrophil releasing active substances. Some data indicated that cathelicidins directly activate neutrophils to mediator release. Human cathelicidin, at a concentration of 20 *μ*g/ml, stimulates neutrophils to the synthesis of proinflammatory CXCL8 under the control of p38 mitogen-activated protein kinase (MAPK) and ERK [47, 48]. It is well known that this chemokine, acting via CXCR2, induces chemotaxis of not only neutrophils but also other granulocytes and stimulates neutrophils to phagocytosis. On the other hand, interesting data suggest that LL-37 inhibits SAA-induced CXCL8 production and causes dramatic inhibition of ERK and p38 MAPK activities [44].

LL-37 is not only involved in regulating the physiological function of neutrophils when they are alive but also can influence neutrophil apoptosis and the physiological function of forming nets after death. LL-37 induces secondary necrosis of apoptotic neutrophils through the increased expression of antiapoptotic protein Bcl-XL and by blocking the activation of caspase-3 [49, 50] via the activation of FPR and purinergic receptor P2X ligand-gated ion channel 7 (P2X7 receptor) on these cells [46]. NETs which are involved in a variety of chronic inflammatory pathologies can release LL-37 in vivo and ex vivo, and LL-37 also promoted peripheral neutrophils to form NETs in a dose-dependent manner ex vivo [51]. And NETs via C1q/LL-37 specifically inhibited interleukin IL-6 secretion by LPS-activated macrophages [52].

### 5.2. Monocytes/Macrophages/Dendritic Cells

Monocytes and macrophages are the immediate arm of the immune system and play an important role in immunomodulatory and tumor immunity via producing both inflammatory mediators and antigen processing. Monocytes are called adult stem cells; it can be differentiated into different cell types: macrophages, DCs, monoosteophils, osteoclast, endothelial cells, etc. For example, monocyte can differentiate into inflammatory macrophages or DCs during inflammation effected by the inflammatory milieu and pathogen-associated pattern-recognition receptors. Macrophages may be characterized as M1- and M2-polarized subtypes; M1 macrophages exhibit inflammatory and antitumor functions via the release of soluble enzyme and cytokines, whereas M2 macrophages have anti-inflammatory functions, may promote tumor cell proliferation, and participate in tissue remodelling. DCs are the most powerful antigen-processing and antigen-presenting cells which can efficiently uptake, process, and present antigens and stimulate the proliferation of nonsensitized cells, be equipped with high phagocytic activity as immature cells and high cytokine-producing capacity as mature cells. It can stimulate the proliferation and activation of non-sensitized T cells, and is the central link to initiate, regulate, and maintain specific antigen induction in vivo. Tumor-specific DC can stimulate specific long-lasting tumor immunity induction.

#### 5.2.1. Monocytes

LL-37 has been shown to be involved in monocyte/macrophage differentiation. It was reported that LL-37 enhances the GM-CSF/IL-4-driven differentiation of blood monocytes into immature DCs [53]. LL-37-derived DCs preserve the basic DC phenotype; LL-37 appropriately promotes maturation of DC and changes the expression of chemokine receptors that facilitate mDC migration to T cell areas. LL-37 in synergy with Peptidoglycan (PGN) can induce monocytes from the peripheral blood of healthy individuals polarized toward the CD14^high^ CD16^+^ subset, and LL-37 further induced PGN-driven differentiated monocytes into immature dendritic cells (iDC), as evident by the increased expression of CD1a, CD86, and HLA-DR markers, resulting in the induction of T cell proliferation and Th17 polarization [54]. It displayed that LL-37 can influence monocyte differentiation, induce PGN-driven monocytes polarized to DCs, and promote proinflammation and adaptive immune response. Other than this, monocytes from the blood sample can differentiate into the population of monocyte-derived bone-forming cells (monosteophils) and accelerate bone repair treating with an effective dose of LL-37 which are uptaken via CXCR2-specific endocytosis of monocytes [55–57].

Monocytes are stimulated by LL-37 to upregulate the release of proinflammatory chemokines (CXCL1, CCL2, and CCL7) and cytokines (IL-8 and IL-6) with IL-1*β* synergistically or not and transcript the genes encoding anti-inflammatory cytokines (IL-10 and IL-19) [58, 59]. Not only upregulate pro- or anti-inflammatory cytokines, LL-37 which inhibit monocytes express some cytokines CXCR2, TNF-*α*, and IL-6 with IL-1*β* synergistically or not. In addition, LL-37 strongly inhibits the synthesis of TNF-*α* and IL-12 by monocytes stimulated with IFN-*γ* [60].

#### 5.2.2. Macrophages

Meantime, LL-37 can regulate the activity of macrophages of the immune system. In macrophages, LL-37 upregulates or downregulates different genes to influence cell functions; the genes predicted to be upregulated by LL-37 were including those encoding chemokines chemokine receptor (CCR) 2, CCL7, IL-8, anti-inflammatory cytokine IL-10, and M-CSF [29]. Contrariwise, this peptide can downregulate another 20 genes, including gene encoding proinflammatory IL-12 [29]. In line with this, cathelicidin and its derivative wildly restrain the production of TNF-*α* and IL-1*β* by IL-32-driven macrophage, promoting to produce the anti-inflammatory cytokine interleukin-1 receptor antagonist (IL-1RA) without changes in chemokine production [61].

While some studies focus on LL-37 regulation of the active macrophages by bacterial components, LL-37 obviously decreases Neisseria meningitides endotoxin lipooligosaccharide (LOS), LPS, or lipoteichoic acid (LTA) which induced both TNF-*α* and nitric oxide (NO) release from macrophages [62–65]. Among them, LOS, LPS, and LTA are bacterial endotoxin. In addition, it was reported that cathelicidin causes significant suppression of producing TNF-*α* by macrophages inducing with arabinosylated lipoarabinomannan (AraLAM). LAM is an important component of the cell wall of *Mycobacterium tuberculosis* [66]. What is more, Hu et al. showed that cathelicidin inhibits pyroptosis of macrophages and proinflammatory cytokine synthesis (IL-1*β*, IL-6, and TNF-*α*) by activated macrophages induced by LPS/ATP in vitro or cecal ligation puncture (CLP) in CLP septic mice; thus, the peptide not only neutralizes the action of LPS but also inhibits the response of P2X7 [67, 68]. Consist with this, exogenous LL-37 decreased TNF-*α* and IL-17 while inducing IL-10 and transforming growth factor *β* (TGF-*β*) production that was also independent of the P2X7 receptor and did not reduce antimycobacterial activity during infection [69]. Thus, LL-37 has a more comprehensive immune regulation function without affecting anti-infection. Interestingly, Ruan et al. uncovered that LTA upregulated the concentration of peptide located mostly in the cytoplasm of macrophages [63]. It indicated that pathogens stimulate body release LL-37 firstly and then exert their anti-infection and immunomodulatory function.

Unlike the proinflammatory and anti-infective functions in an inflammatory environment, LL-37 can overexpress in certain tumors and promote the differentiation of macrophages to M2, which in turn promotes tumorigenesis. As cathelicidin of mice, CRAMPs are proved to promote tumorigenesis via macrophage. With prostate cancer CRAMP^(+)^, more immature myeloid progenitors (IMPs) polarize into protumorigenic M2 macrophages than CRAMP^(−)^ TME. Meanwhile, in vitro experiments confirmed that CRAMP can mediate autocrine signaling to promote M-CSF and monocyte chemotactic protein (MCP) 1 overexpressing in TRAMP-C1 cells (prostate cancer cell lines without CRAMP expression) to regulate macrophage differentiation to M2 by activating STAT3 [70]. Furthermore, the human cathelicidin is proudly expressed by tumor-associated macrophages (TAMs) present within the pancreatic ductal adenocarcinoma (PDAC) stroma and induced by CSC-secreted tumor growth factor-*β* (TGF-*β*) family members Nodal and ActivinA. The synthesis human cathelicidin enhances the ability of pancreatic cancer stem cell (CSC) to invasion, self-renewal, and tumorigenesis, via the G protein-coupled receptor (GPCR), FPR2 and P2X7 receptor [71]. Thus, LL-37 as a tumor microenvironment factor plays a critical role in tumorigenesis. Similarly, human macrophages were cocultured with colorectal cancer cells SW480 or HCT116 to mimic the tumor microenvironment; the mimic TAMs enhance the production and release of antimicrobial peptides LL-37 to promote the proliferation of colorectal cancer cells via the Wnt/*β*-catenin pathway [72].

Different environments or stimuli can affect the function and differentiation of monocytes/macrophages. And LL-37 plays two opposite roles in it. In an inflammatory or infective environment, LL-37 can promote the proinflammatory and anti-infective functions of monocytes/macrophages, while also carefully monitoring the trend of inflammation, promoting the synthesis of anti-inflammatory factors, preventing the situation from expanding, and maintaining the overall immune balance of the body. Tumor-derived LL-37, an overexpressed peptide in the tumor environment, helps tumor cells to polarize macrophages into M2 type of TAMs, inhibit immune function, and promote tumorigenesis.

#### 5.2.3. Dendritic Cells

As a potent modifier of DC differentiation, LL-37 is bridging innate and adaptive immune responses at sites of inflammation, where high levels of LL-37 secreted by recruited neutrophils and resident epithelial cells chemoattract pre-DC. The addition of peptide suppressed mature DC (stimulated by LPS) release of IL-6 and TNF-*α*; the expression of HLA-DR, CD80, CD83, and CD86; and the chemokine receptor CCR7. This suppression is a concentration-dependent manner; up to 20 *μ*g/ml of LL-37 will result in a total inhibition of secretion [73]. Not only for DCs but also influence for T cells via DCs, DC exposure with LL-37 and LPS resulted in a prominently reduced capacity of DCs to stimulate CD4^+^ T cells, as decreased IFN-*γ* and IL-2 secrete and their proliferation [73]. On the contrary, another study using LPS stimulate DCs derived from monocytes by incubation with IL-4, GM-CSF, and LL-37; the results displayed upregulated endocytic capacity, modified phagocytotic receptor expression and function, upregulated costimulatory molecule expression, enhanced the secretion of T helper cell (Th) 1 cytokines, and then promoted Th1 responses in vitro [53]. In like manner, it is demonstrated that the addition of LL-37 (without LPS or other antigen) is internalized by immature dendritic cells derived from human peripheral blood monocytes (MDDC) with subsequent localization primarily in the cytoplasmic compartment and then could also be transported into the nucleus of MDDC, caused phenotypic changes, and characterized by an increased expression of the antigen-presenting molecule HLA-DR and the costimulatory molecule CD86 [74]. Above data look like in contradiction, but the sequence of stimulation between LL-37 and LPS is different. LL-37 can restrict the proinflammation of LPS and control immunity development. Addition to this, LL-37 can influence the function of DCs by internalization and manipulate T cell polarization by DC. Taken together, these findings suggest that, after triggering of the innate immunity, LL-37 would be released to affect sequent cellular adaptive immune.

Besides itself of LL-37, Hurtado and Peh suggested that LL-37 can bind to bacterial DNA (CpG oligodeoxynucleotides) which brings about a significant reduction in the time through sensing the presence of bacterial DNA via TLR9 by B cells and plasmacytoid DCs (pDCs) [75]. Since LL-37 is a cationic charge, a stable LL37/nucleic acid complex is formed. The latter enters the endosomal compartment of pDCs and stimulates TLR9 and TLR7. LL-37 combine with extracellular autonuclei to form an effective trigger for the release of IFN from pDCs [76]. Furthermore, LL-37/RNA complexes can also induce the activation of IFN-conditioned myeloid DCs (mDCs) by TLR7/8 stimulation [77]. Thus, these results proved that LL-37 play a crucial role in the formation of psoriasis.

### 5.3. Lymphocytes

Lymphocytes include T cells (thymocytes), B cells (bone marrow or bursa-derived cells), and NK cells. T cells and B cells are mainly involved in the adaptive immune system. The primary T cells migrate within the secondary lymphoid organs where they encounter and interact with the DCs and further differentiate into T cell subsets. For example, some T cells (also known as CD4^+^ cells) called Th cells can produce cytokines that direct immune responses, for example, Th1, Th17, and Th2. Th1 cells are differentiated induced by cytokines such as IL-12; secrete IL-2, IFN-*γ*, TNF*β*, and other cytokines; and participate in regulating cellular immunity, assist in cytotoxic T cell differentiation, and participate in delayed-type hypersensitivity reactions, while Th17 is differentiated under the stimulation of IL-6 and IL-23 and mainly secretes IL17, IL1, IL-6, and TNF-*α*. These cytokines can collectively mobilize, recruit, and activate neutrophils; thus, like Th1, polarized Th17 cells have the capacity to cause inflammation and autoimmune disease. Th2 helper cells are mainly immune responses against extracellular multicellular parasites, which are mainly induced by IL-4, and mainly secretes IL-4, IL-5, and IL-10. Regulatory T cell (Tregs) was named as suppressor T cell in the 1970s. Tregs is a key cell in the negative regulation of the body's immune response, playing a paramount role in maintaining self-tolerance and immune homeostasis and participating in tumor cells to escape the body's immune surveillance [78]. There are many types of Tregs, such as CD4^+^ CD25^+^ T cells. With the help of activated Th cells and antigen-presenting cells (APCs), B cells become activated B cells by antigen stimulation and then differentiate into plasma cells to synthesize and secrete various antibodies, mainly performing humoral immunity of the body. NK cells are part of the innate immune system that distinguishes between abnormal cells (infected cells and tumor cells) and normal cells by recognizing changes of molecules known as Major Histocompatibility Complexes (MHC) class I on the cell surface and via releasing cytotoxicity (cells-killing) granules which then destroy the altered cells and plays an important role in protecting the host from tumor and viral infections.

#### 5.3.1. T Cells

Early research shows LL-37-derived mDC maturation with LPS produces a characteristic Th1-inducing cytokine profile (significantly an increase of IL-12, IL-6, and TNF-*α* and significantly a decrease of IL-4). Furthermore, significantly increased synthesis of IFN-*γ* by T cells was tested which is stimulated by LL-37-derived mDC. Thus, LL-37 appears to act as a bridge between the innate and adaptive immune systems [53], while LL-37 not only produces an enhanced Th1 response but also produces an adjuvant that enhances the Th17 response in the oral mucosa, where mFPR2 on M cells interacts with LL-37-Ag and is recognized by APC near the M cell, mature to CD11c^+^CD70^+^ APC, which subsequently produces a Th17-biased environment by increasing IL-17, and leads to an increase in the formation of germinal centre (GC) B cells and GC; thus, LL-37 mediates an Ag-specific immune response through regulating the mucosal immune environment [79]. Interestingly, by assessing the role of LL-37 in peripheral blood mononuclear cells (PBMC), the researchers found that LL-37 also promotes the production of regulatory T cells, while LL-37 does not affect T cell activation; in the context of inflammation (PHA activation), peptides can induce resting T cell proliferation, significantly increasing Tregs production and decreasing proinflammatory factor expression (INF-*γ*, TNF-*α*) of PBMC; indicating that when the peptide plays its own anti-infective property, the control proinflammatory responses are always accompanied, in order to protect the body against severe inflammatory response [80]. These results show the two sidedness of antimicrobial peptides. In the face of different immune cell populations and different microenvironments, antibacterial peptides will exhibit different states, even diametrically opposite immune responses, so the microenvironment obviously plays a pivotal role in determining how T cells respond to LL-37.

#### 5.3.2. NK Cells

As an important part of the innate immune system, NK cells can be used for immune surveillance of certain tumor and virus-infected cells. Cathelicidin was observed to be abundant in tumor-infiltrating NK1.1^+^ cells in mice. Functional in vitro analyses found that NK cells derived from cathelicidin knockout mice (Camp^−/−^) versus wild-type mice showed impaired cytotoxic activity toward tumor targets. Moreover, Camp^−/−^ permitted faster tumor growth than wild-type controls in two different xenograft tumor mouse models (murine B16 melanoma and RMA-S lymphoma) that exclude an observed perforin deficiency [16]. The findings indicate the significance of cathelicidin to NK cell function and in vivo tumor defense. In addition, LL-37 improves CpG delivery to intracellular TLR9 results in the enhanced proliferation and activation of NK cells, to prevent relapse in the case of ovarian cancer [81]. However, the details of mechanism that LL-37 interact with NK cells are needed to be clarified further.

### 5.4. Mast Cells

Mast cells are an important component of host defense pathogens and can affect both innate and acquired immune responses. Mast cells participate in the entire process of inflammation, such as promoting inflammation and limiting inflammation, through the production of mediators, including cytokines, chemokines, and biologically active mediators. Various endogenous and exogenous mediators can activate cells through different receptors expressed by mast cells, and activated cells rapidly release relevant mediators stored in cytoplasmic granules to participate in inflammatory responses such as histamine, protease, and cytokines (IL-3, IL-4, IL-6, IL-8, IL-10, TNF-*α*, etc.) [82, 83]. In addition, activated mast cells can release a variety of newly produced lipid mediators including leukotriene (LT), prostaglandin (PG), thromboxane (TX), and platelet-activating factor (PAF) [82, 84].

In mast cells, this host-defense peptide causes degranulation assessed by histamine or *β*-hexosaminidase release by intracellular Ca^2+^ mobilization [34, 85] but also the production and release cytokines (IL-1*β*, IL-2, IL-4, IL-6, IL-31, TNF-*α*, and GM-CSF) and chemokines (CCL 2 and CCL3) stimulated with 10 *μ*g/ml LL-37 for 3-24 h [34] or 5-20 *μ*g/ml LL-37 for 6 h [86] in a dose-dependent and time-dependent fashion [34, 86]. In addition, this peptide activates mast cells to produce strong proinflammatory mediators (LTC4 and PGD2) [86]. These factors play a critical role involved in inflammatory and anti-inflammatory responses. In line with this, LAD2 cells were treated with 1 *μ*g/ml cathelicidin which tended to increase the level of TLR4 expression, Th1 cytokines IL-2, proinflammatory cytokines TNF-*α* and IL-1*β* and significantly induced Th2 cytokines, IL-4 and IL-5 release; however, significantly Th2 cytokines could be inhibited by LPS, although IL-1*β* production was not diminished [87]. Thus, these data reveal that LL-37 synergism the bacterial components may skew the mast cell toward innate immunity and adaptive immunity [87].

While studying the functional effects of LL-37 on mast cells, some literature also explored and described its mechanism of action. LL-37 may bind the negatively charged cell surface molecules, rapidly internalize into the cells via clathrin-mediated endocytosis, and interact with Mas-related gene X2 (MrgX2) to activate mast cell (LAD2 cell) degranulation and release of de novo synthesized mediator function primarily; this effect is associated with the activation of the Gi protein, PLC/PKC/Calcium/NFAT, PI3K/Akt, and MAPK signaling pathways [34, 88, 89]. Notwithstanding the foregoing, LL-37 (10 *μ*M) enable to permeabilize both nuclear and plasma membranes to enhance the export of nucleic acids of mast cells, total protein, and lactate dehydrogenase (LDH) [90]. Thus, it was proposed that LL-37-induced release of nucleic acids from mast cells may be another mechanism of LL-37 moderating the immune response.

### 5.5. MSCs

MSCs are pluripotent stem cells that share all the commonalities of stem cells, namely, self-renewal and multidirectional differentiation. MSCs regulate the innate immune system and adaptive immune system function through direct contact between cells and secretion of mesenchymal stem cells. MSCs can induce immunomodulatory effects on various cells associated with carcinogenesis via producing a variety of cytokines and growth factors [91, 92].

LL-37 augments the promoting tumorigenesis properties of MSCs by recruiting them to ovarian tumors through FPR2 [37]. Follow-up researches in vitro show MSCs, after 48 h of LL-37 treatment, were stimulated to release significantly more angiogenic and inflammatory molecules compared with untreated cells, including IL-1 receptor antagonist, IL-6, IL-10, CCL5, vascular endothelial growth factor (VEGF), and matrix metalloproteinase-2 (MMP-2) [37, 93]. Besides LL-37 treatment enhanced the proliferation and migration of human adipose-derived stromal/stem cells (ASCs), it also promotes expressing FPR2, early growth response (EGR) 1 expression, and MAPK activation, and that preconditioning of ASCs with LL-37 has a strong potential to promote cell proliferation, cell migration, and paracrine actions, which may be useful in terms of implantation for tissue regeneration [37]. However, research in vivo was indicated that LL-37-mediated recruitment of MSCs can also facilitate ovarian tumor progression through secreting proangiogenic factors which resulted in a significant increasing number of vascular channels in nude mice and some cytokines including IL-1*β*, IL-6, IL-8, IL-10, and TNF-*α* (and the reduction of IL-12 expression). Consistently, in vitro endothelial cell formation by MSCs is enhanced by LL-37 presence with a positive effect on tumor growth [37]. Moreover, LL-37 modulates TLR3 expression, promotes higher levels of anti-inflammatory factors (indoleamine2,3-dioxygenase (IDO), IL-10, and TGF-*β*), and boosts the suppressive function of pMSCs over stimulated T cells; thus, LL-37 may offer protection against opportunist microorganisms, meanwhile ensuring the maintenance of MSCs in their highest anti-inflammatory state [94]. Therefore, the LL-37 boost proliferation, immunosuppressive, and migratory potential of MSCs to promoting tumorigenesis or anti-inflammation.

## 6. Diseases

Defects in the expression or processing of immunomodulatory peptide, resulting in abnormalities in immune regulation, lead to inflammation-related diseases such as inflammatory bowel disease (IBD), psoriasis, periodontal disease, or cancers. Thus, detailed knowledge about the associated molecular mode of action on tissues and their various cells is necessary to understand the pathogenesis of these diseases.

### 6.1. Inflammatory Bowel Disease

IBD, including UC and CD, is an idiopathic enteritis disease involving the ileum, rectum, and colon. It is unclear about the etiology and pathogenesis [83]. As far as we know, an abnormal reaction of the intestinal mucosal immune system results in inflammatory response of IBD. It is difficult to cure using current treatments, and new therapies are needed. More than one document has reported increased expression of LL-37 on the intestinal mucosa of patients with IBD. Moreover, this high expression occurs simultaneously in the inflammatory and uninflamed colonic mucosa of UC patients [95]. Nowadays, the regulatory mechanism of LL-37 induction was investigated that in human colonic SEMFs, the expression of LL-37 upregulating was probably induced by TLR-3 stimulation via poly(I:C) [20, 96]. Then, the increasing complex of LL-37-bacDNA may further promote more expression of LL-37 in primary human monocytes by activating the TLR9-ERK1/2 pathway and the differentiation of T cells towards Th1, Th2, and Th17 to huge scope inflammation [97–99]. Perhaps precisely because of this, bacteria may make a milieu by releasing bacDNA to utilize and resist host antimicrobial peptides as a “trojan horse” in IBD to evade immune elimination [99]. Moreover, LL-37 levels may be a marker to reflect intestinal stricture in CD patients, low levels presage a significant elevated risk of intestinal stricture, and high levels relate to good prognosis [98, 100].

Although current studies show that LL-37 appears to be an accomplice of bacterial mucosal inflammation in the etiology of IBD, there are still many studies that show that LL-37 is a new direction for the treatment of IBD. In the inflamed mucosa of IBD, LL-37 still might exert antibacterial and neutralization of LPS activities to defend the intestine from pathogen invasion and superabundance inflammation [20]. This result is consistent with other reports that administration of cathelicidin and analog is effective in UC and CD models. mCRAMP (an analog of LL-37) could attenuate dextran sulfate sodium- (DSS-) induced colitis in a murine model and relieved neutrophil infiltration in colitis tissues [101]. Cathelicidin-BF (C-BF), a snake cathelicidin-derived antimicrobial peptide, which has antibacterial activity, mitigates inflammation and ameliorates damaged barrier of DSS-induced ulcerative colitis in vivo via inhibited phosphorylation of NF-*κ*B (p65) [102, 103]. Moreover, a short-term treatment with 2000 IU/day vitamin D significantly increased 25(OH)D levels in blood which facilitate to elevate the level of LL-37 to exert immune-modulatory and anti-inflammatory effects to prolong remission in CD [104].

Recently, cathelicidin gene and/or recombinant protein therapy for UC and CD seems to be popular. The mCRAMP-encoding plasmid may reverse increased levels of cytokines and apoptosis, promote mucus protein expression and secretion, and prevent ulcerative colitis by regulating inflammation and mucus secretion in exacerbated colitis cnlp^−/−^ mice through the intrarectal administration [105]. Another study oral administration of mCRAMP-transformed *Lactococcus lactis* effectively produce mCRAMP and alleviated the degree of inflammation reflected by the decrease of the number of apoptotic cells, myeloperoxidase activity, and malondialdehyde level; then, the clinical symptoms were improved, crypt integrity is maintained, and the mucus content is preserved [106]. Additionally, injection of cathelicidin-overexpressing lentiviruses induced collagen expression to efficiently attenuate colitis-associated intestinal fibrosis through inhibiting transforming growth factor-1 (TGF-1) and IGF-1 [107]. Therefore, cathelicidin might be useful for patients with IBD via regulating the intestinal mucosal immune system. LL-37 seems to be both morbific and treatable; as mentioned above, the reasons might be the microenvironmental impact or the difference between endogenous or exogenous; however, the true face of LL-37 needs further exploration.

### 6.2. Psoriasis

Psoriasis is a long-lasting autoimmune, chronic inflammatory skin disease; disturbances in the innate and adaptive cutaneous immune responses lead to uncontrolled keratinocyte proliferation and dysfunctional differentiation, characterized by patches of abnormal skin [108]. Many studies have shown that psoriasis is associated with abnormal expression and activity of cathelicidin.

Even though the exact role of LL-37 in the pathogenesis of psoriasis remains unclear, it was found LL-37 were significantly increased in psoriatic plaques and LL-37 play an important role in psoriasis. LL-37 can affect keratinocyte, activate innate and adaptive cutaneous immune responses, and maintain the autoinflammatory cascade [109]. It was indicated that cathelicidin not only induces keratinocyte migration and proliferation [110] but also stimulates keratinocytes to release different effect cytokines (including IL-1*β*, IL-6, IL-18, IL-20, and GM-CSF), chemokines (i.e., CCL2, CCL5, CCL20, CXCL8, and CXCL10), and anti-inflammatory cytokine IL-10 via EGFR, G protein, and PLC signaling pathways [110–112]. In addition, LL-37 enhance UV-induced IL-1*β* secretion and inflammasome activation via acting on the P2X7 receptor on keratinocytes [113].

Both LL-37 or the complex LL-37 and nucleic acid regulate immune responses in psoriasis. LL-37 stimulate mDCs to secrete TNF-*α* and IL-6, and mDCs are able to activate naïve-T cells and induce their polarization to Th1/Th17 cells in psoriasis [53, 79, 114, 115]. Meanwhile, LL-37 isolated from lesioned psoriatic skin scavenges can form complexes with human self-nucleic acid from dying cells. The LL-37/self-DNA complexes are sensed by dermal pDCs via endocytosis and stimulate IFN-*α* response via the TLR9/MyD88/IRF7 signaling pathway [116, 117] whereas LL-37/self-RNA complexes can activate TLR7 to release IFN-*α* [77]. Then, large amounts of IFN-*α* and activated pDCs and mDC activate downstream self-reactive T cells, which mediate immune responses and result in psoriatic lesion formation [76, 117]. Thus, LL-37 converts inert self-nucleic acid into a potent trigger of interferon production by pDCs in psoriatic skin [76].

Interestingly, researcher found that two-thirds of patients with moderate-to-severe plaque psoriasis harbor CD4^+^ and/or CD8^+^ T cells specific for LL-37 infiltrating lesioned skin, which produce IFN-*γ* (Th1 cytokines), and CD4^+^ T cells also produce Th17 cytokines (IL-17, IL-21, and IL-22) [23]. The subsequent silico docking study further predicted the high binding affinities of multiple 9-mer peptides derived from LL-37 to the *HLA-C*^∗^ 06:02 molecule to propose a mechanism of the interaction between this complex and T cells via TCRs LL-37-*HLA- C*^∗^ 06:02 [118]. Thus, this study provides evidence for a role of LL-37 in psoriasis.

Besides DCs and T cells, polymorphonuclear leukocytes (PMNs) are abundant in psoriatic skin and are primary sources for LL-37. The human and bacterial RNA complexed with LL-37 not only stimulate PMNs from psoriasis patients that respond via TLR8 by producing TNF-*α*, IL-6, IL-8, and IL-1*β*, and NET-release; they also can be released by PMNs. The same complex and complex RNA-LL-37 were found to be highly abundant in PMNs from psoriasis patients compared to PMNs from healthy donors [119, 120]. Moreover, RNA-LL37-induced NETs propagated PMN activation and could thus fuel a PMN-mediated and self-sustaining inflammatory loop that may represent an unexpected early initiator or amplifying event in psoriasis. Therefore, in psoriatic lesions, RNA-LL37-driven PMN activation may contribute to a vicious cycle of inflammation and immune cell attraction [121].

### 6.3. Periodontal Diseases

Periodontal disease refers to the disease that occurs in periodontal tissue, including gingival disease with inflammation only involving gingival tissue and periodontitis involving deep periodontal tissue (periodontal membrane, alveolar bone, and cementum). Periodontal disease is a common oral disease, which is the main cause of tooth loss in adults. It is a refractory disease, which cannot be cured for a long time, and is easy to develop into chronic. The main clinical manifestations are alveolar bone absorption, periodontal bag formation, gingival bleeding and inflammation, tooth loosening, and so on. This kind of lesions is caused by dysregulation of microbiota-host homeostasis of oral cavity which can give rise to inflammation and bone loss [121].

Salivary glands, oral mucosa, and immune cells in the oral cavity can express this kind of peptide [122]. Some studies found that some diseases (like Kostmann syndrome, periodontal disease-associated bacteria, and chronic periodontitis) in humans are related to the aberrant level of cathelicidins [123–126]. Patients with Kostmann syndrome often have low levels of LL-37 in serum and saliva because of deficiency of neutrophils, with severe alveolar bone loss or even periodontal ligament infection [124, 125]. About a third of the aggressive periodontitis patients lack active cathelicidin in the gingival crevicular fluid [123]. As antimicrobial peptides, of course, inhibiting the growth of various periodontal bacteria (*Porphyromonas gingivalis*, *Fusobacterium nucleatum*, *Treponema denticola*, and *Aggregatibacter actinomycetemcomitans*) to keep the microbiota-host homeostasis is just one function of LL-37 [127, 128]. In oral cavity and the skin, as we described previously in this review, the interaction between TLR ligands (as LPS and flagellin), self-DNA or self-RNA, and LL-37 may be involved in infection and inflammation [127, 128]. On the other hand, TLR ligands induce receptor activator of nuclear factor kappa-B ligand (RANKL) expression in osteoblasts and TNF-*α* production in BMMs. RANKL binds to RANK expressed in osteoclast precursors and subsequently induces osteoclast differentiation [129, 130]. Mature osteoclasts also express RANK and TLR4 [131] and promote the bone-resorbing activity of osteoclasts through TRAF6 which is a common downstream molecule [131–133], while TLR ligands also induce LL-37 expression in several different host cells including osteoblasts and immune cells. Thus, LL-37 can inhibit TLR ligands that induced inflammation and bone loss through antimicrobe and neutralize LPS and flagellin.

However, beyond that, Kittaka et al. found for the first time that LL-37 can regulate angiogenesis and the recruitment of stem cells to promote bone regeneration [134]. It is observed that morphologically fibroblastic cells with STRO-1^+^ (a marker of MSCs), at an early stage of tissue regeneration in a rat suffering from calvarial bone defect treated with cathelicidins, accumulated in the bone defect area where endothelial cells were also localized. Recently, Yu et al. further proved these findings. It is found that LL-37 promoted bone marrow stromal cell (BMSC) proliferation, migration, and osteogenic differentiation within normal and inflammatory microenvironments via P2X7 receptor and MAPK signaling pathway and can inhibit inflammation, markedly inhibiting osteoclastic bone resorption through P2X7 receptor and MAPK pathway [135].

As we all know, not only limited to the oral cavity, LL-37 plays a very important role in the promotion of bone repair in the bone-related diseases especially for inflammation-induced bone loss, osteoporosis, bone fracture, and so on. Zhang et al. first showed that LL-37 entered monocytes from blood source through the endocytosis of CXCR2 and promoted its differentiation into novel bone-forming cells (monosteophils) [55, 56]. Furthermore, Zhang et al. confirmed by experiments in vivo that LL-37 can promote bone repair in an animal model of bone injury by inducing monocytes to human monoosteophils, characterized as CD45^+^*α*3^+^*α*3*β*^+^CD34^−^CD14^−^BAP (bone alkaline phosphatase)^−^ cells [57]. At the same time, Supanchart et al. also confirmed that LL-37 inhibits the in vitro osteoclastogenesis via preventing nuclear translocation of NFAT2 (the main switch of osteoclast differentiation and bone resorption) by inhibiting the calcineurin activity [136].

At present, the study demonstrates that LL-37 can be a potential candidate drug for promoting osteogenesis and for inhibiting bacterial growth and osteoclastogenesis. Liu et al. proved this peptide significantly promotes MSC differentiation, migration, and proliferation, inhibiting LPS-induced osteoclast formation and bacterial activity in vitro; so, LL-37 combined with bone morphogenetic protein 2 (BMP2) can regulate MSCs to promote calvarial repair in an osteolytic model [137]. And LL-37 had been used as modification of bone implants to optimize osteointegration ability. He et al. loaded LL-37 on Ti substrates that benefited the cell viability, recruitment, and paracrine responses of MSCs and macrophages in vitro and induced MSC and macrophage recruitments to injury sites, and the inflammatory response was positively regulated, facilitated bone formation, and improved osteointegration via the regulation of physiological functions of MSCs and macrophages in vivo [138, 139]. These studies provide a promising strategy in the design of bone repair-related oral and bone-related diseases.

### 6.4. Cancer

Last few years, some evidence from cancer biology studies indicates that human cathelicidin is involved in carcinogenesis. As noted above, this peptide expression is out of control and dysregulated in some cancer types. For example, LL-37 is upregulated in various ovarian tumor subtypes, compared with normal ovarian tissues [140]. In these tumors, LL-37 has been shown to promote tumor progression through its influence on mesenchymal stromal/stem cells [37, 94, 141] via some activated pathways, such as FPR2 [142, 143], IGF-1receptor and ErbB2 [144, 145], and CXCR4 [146]. At the same time, LL-37 also promotes tumor formation by affecting immune-active cells. The interaction of LL-37 with TAM is mentioned above. Overexpressed LL-37 in tumors promotes the differentiation of macrophages to M2, which in turn promotes tumorigenesis. Furthermore, the human cathelicidin is significantly expressed by TAMs present, which promote the proliferation of colorectal cancer cells. Thus, LL-37 as a tumor microenvironment factor plays a critical role in tumorigenesis. Similarly, LL-37 augments the promoting tumorigenesis properties of MSCs by recruiting them to ovarian tumors and enhancing their proliferation and migration, facilitating tumor progression through secreting proangiogenic factors and some cytokines resulted in a significant number of vascular channels and immunosuppressive.

The antitumor activity of LL-37 may be linked to its role to mediated apoptosis and as an immunomodulatory agent. In colon cancer, studies indicated that LL-37 suppressed tumor development through different pathways. Ren et al. suggested that LL-37 inhibited colon cancer by the activation of a GPCR-p53-Bax/Bak/Bcl-2 signaling cascade that triggers AIF/EndoG-mediated apoptosis, rather than caspase-dependent apoptosis [147]. Cheng et al. found that LL-37 inhibited colon cancer development through indirect pathways, which include interference with epithelial-mesenchymal transition of colon cancer cells and suppression of fibroblast-supported colon cancer cell proliferation [148]. As an immunomodulatory agent of LL-37, studies also have shown that this peptide enhanced the sensing of CpG oligodeoxynucleotides by immunocompetent cells (B cells, pDCs, and NK cells), and these CpG oligodeoxynucleotides enhance the antitumor activity through affecting TLR9 [140] and induce IFN-*γ* expression, proliferation, and activation of NK cells in treated tumors [81]. Furthermore, LL-37 induced an activation and expansion of OVA-antigen-specific CD8^+^ T cells in draining lymph nodes and the tumor microenvironment [149]. This process was associated with delay in tumor growth, while preclinical studies have also demonstrated that intratumoral injections of LL-37 stimulate the innate immune system by the activation of pDCs [150]. These cells can induct and maintain antitumor immune responses and mediate tumor destruction [151]. These findings suggest that LL-37 could induce antitumor immunity and provide a promising strategy for immunotherapy.

## 7. Conclusions

**Figure 1:**
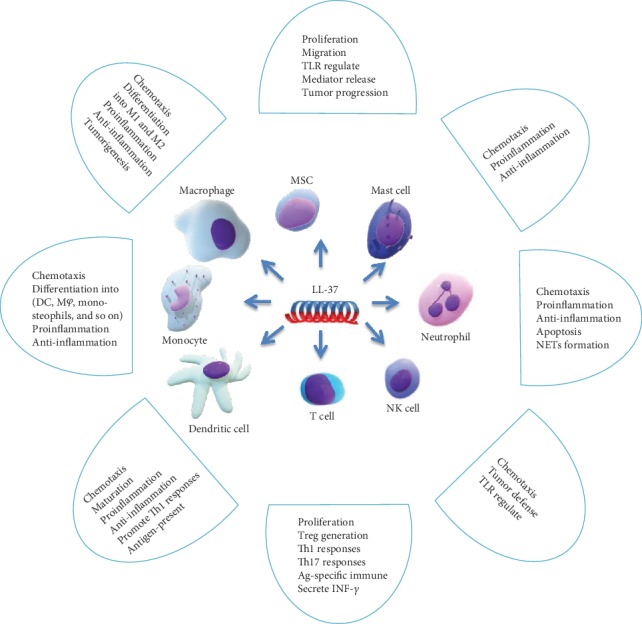
Immunomodulatory function of LL-37.

Recent work has conclusively demonstrated that human cathelicidin LL-37 represents a chemical defense as an essential component of innate immunity that eliminates invading pathogens and restores homeostasis. In addition to its antimicrobial activities, accumulated evidence reveals pleiotropic functions of LL-37 that influence immune responses (see Figure 1). The immunomodulatory function of LL-37 has its two sides. On the one hand, LL-37 can chemotactically activate cells to the infected or abnormal parts of the body and can synergize with other active substances to promote the differentiation of immunocompetent cells into proinflammatory cells that promote immune responses; for example, monocyte can be further differentiated into macrophages or DCs; macrophages can differentiate into M1-type cells, and DCs can further mature to present more antigen; T cells can differentiate into Th1, Th17 type cells, etc.; LL-37 can stimulate immunocompetent cells to secrete proinflammatory cytokines, chemokines, costimulatory factors, cellular receptors, etc., promote immune response, exert its anti-infective and antitumor effects, bridge innate immunity and adaptive immunity, and promote the responses of Th by DCs to start up the secondary immune system. On the other hand, LL-37 also has the ability to inhibit inflammation and promote carcinogenesis. This peptide can augment the release of anti-inflammatory cytokines, neutralize bacterial LPS, inhibit the release of proinflammatory factors, limit the expansion of inflammation, maintain the body's immune balance, and at the same time, recruit MSCs into tumors to enhance the immunosuppressive effect; in the tumor environment, LL-37 facilitate macrophage differentiation to anti-inflammatory M2-type cells, along with MSCs exerting carcinogenesis.

**Figure 2:**
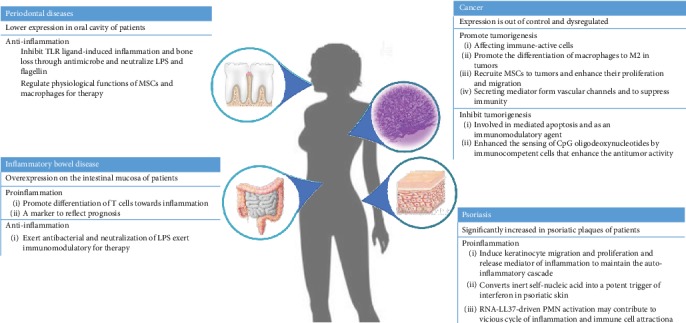
The roles of LL-37 in the diseases.

Under normal circumstances, LL-37 can help the body maintain homeostasis, but once this steady state is broken, LL-37 can become a disease-causing factor (see Figure 2). In IBD, psoriasis LL-37 is overexpressed. In the case, LL-37 promotes the progression of inflammation, destroying the body's homeostasis and causing autoimmune disease. In tumors, overexpressed LL-37 can affect MSCs and macrophage to promote tumor cell growth and promote tumorigenesis. Due to the ubiquitous expression in different anatomical sites, the production of LL-37 appears to be regulated in a tissue- or even cell-specific manner. The insufficient expression of LL-37 may increase susceptibility to infections and inflammation, such as periodontal diseases; the patients with severe alveolar bone loss or even periodontal ligament infection always accompany with low levels of LL-37 in serum and saliva. Further advances in understanding the biological activity of LL-37 will create an attractive target for therapeutic intervention in infectious and inflammatory diseases.
